# Exploring the Interconnections of Functional Gut Disorders and Inflammatory Bowel Disease: A Narrative Review Article

**DOI:** 10.7759/cureus.53699

**Published:** 2024-02-06

**Authors:** David Huynh, Myat Myat Khaing

**Affiliations:** 1 Gastroenterology and Hepatology, The Prince Charles Hospital, Brisbane, AUS

**Keywords:** and psychological, diagnostic, gastrointestinal health, inflammatory bowel disease (ibd), functional gut disorders

## Abstract

This review reveals details of the interaction between disorders of gut-brain interaction (DGBI) and inflammatory bowel disease (IBD) by providing an in-depth review of that relationship. The review provides a nuanced understanding of this multifaceted dynamic by spanning shared symptomatology, the impact of inflammation on functional aspects, and addressing diagnostic challenges, psychological influences, treatment strategies, and emerging research directions. By synthesizing current knowledge and identifying gaps in understanding, this article aims to contribute to the evolving discourse surrounding the interplay between IBD and DGBI, offering valuable insights for clinicians, researchers, and healthcare professionals navigating the complexities of gastrointestinal health.

## Introduction and background

Disorders of gut-brain interaction (DGBI) have garnered significant attention from researchers, leading to a deeper understanding of the medical situation and improved support for its care through ongoing studies. DGBI constitutes a category of gastrointestinal disorders characterized by disturbances in gut function without apparent structural or biochemical abnormalities. Unlike organic disorders with identifiable pathology, functional gut disorders primarily present as a constellation of symptoms related to the motility, sensitivity, and function of the gastrointestinal tract [1,2]. This disorder is centered around patient symptomatology rather than observed biochemical or structural abnormalities, making diagnosis and management challenging. Therefore, it is essential to rule out recognized gastrointestinal disorders before reaching a diagnosis [1].

Health conditions falling under the umbrella of DBGI include irritable bowel syndrome, functional bloating, and functional dyspepsia [2]. IBD encompasses conditions like Crohn's disease and ulcerative colitis [3]. The significant symptom overlap in these diseases can complicate diagnosis, with affected individuals experiencing discomfort, bloating, irregular bowel movements, and stomach pain. Contributing causes to these symptoms often include stress and nutrition [2-4]. Understanding these conditions becomes crucial for developing effective management strategies, given their substantial prevalence and impact on a noteworthy segment of the population. Improving diagnostic precision and formulating targeted therapeutic strategies depend on unraveling the multifaceted nature of DGBI and IBD [5-10].

Acknowledging shared symptomatology and diagnostic intricacies lays the foundation for a holistic examination of how these dissimilar entities converge in the context of gastrointestinal health. As we delve into the details of shared symptoms, we address the diagnostic challenges clinicians face when distinguishing between these conditions, emphasizing the need for a unique approach to patient care. To guide the reader through our exploration, this section outlines the key objectives of the review, including deciphering the interplay between chronic inflammation and functional aspects of the gut, navigating shared symptomatology, and addressing challenges in accurate diagnosis. This section emphasizes the imperative for a comprehensive understanding of the interplay between these two distinct yet interconnected aspects of gastrointestinal health. We aim to clarify the scope and focus of the comprehensive understanding sought in the subsequent sections.

## Review

Potential DGBI as precursors to IBD

There is research that suggests that DGBI might be a risk factor or predisposing factor for the development of IBD. This intriguing connection underscores the intricate nature of the interplay between the DGBI and the development of chronic inflammatory conditions in IBD, mainly dysbiosis related to chronic inflammation [7,11-14]. A potential theory is that the symptoms that relate to DGBI could represent chronic subclinical inflammation related to IBD that would later manifest in a different phenotype that would be more in keeping with a clinical picture of IBD [8,15,16]. With chronic inflammatory changes to the gut, there will be altered gastrointestinal motility, microbiome dysbiosis, impaired intestinal permeability, immune-system activation, and visceral hypersensitivity, all overlapping the DGBI pathophysiology theory [16,17].

Disorders of gut-brain interactions

DGBI are chronic gastrointestinal symptoms (abdominal pain, dyspepsia, diarrhea, abdominal bloating, and constipation) that occur in the absence of demonstrable pathology or objective etiology [1]. The current classification system (ROME IV) divides this into 33 adult disorders and 20 pediatric disorders, the most common being irritable bowel syndrome. DGBI is very common worldwide, with a prevalence of 40%, being more common in women than men, and they account for 30% of gastroenterology outpatient consultations. This condition is often accompanied by other related chronic health conditions such as fibromyalgia, metabolic disorders, chronic fatigue syndromes, and mental health conditions such as depression and anxiety [11,14]. It is also seen in other organic chronic health issues such as chronic kidney disease and heart failure, as this can come with psychological issues that could lead to DGBI. Of patients with quiescent IBD, approximately 25% will have DGBI [15,16]. 

Pathophysiology of overlap with DGBI and IBD

IBD, characterized by continual gastrointestinal tract irritation due to an immune response, involves genetic predispositions, environmental factors, and immune system dysregulation. Studies on the gut microbiota and environmental/geographical changes have shown modifications in microorganism composition, triggering the inflammatory cascade in IBD [15,17,18]. Ultimately, IBD is marked by structural damage, including mucosal ulcerations and transmural inflammation, contributing to the clinical manifestations and complications. This known mechanism overlaps considerably with the speculated pathophysiology of DGBI [19].

In the context of IBD, post-inflammatory changes in IBD can lead to alterations in gut motility, permeability, impaired colorectal function, and visceral hypersensitivity [18,19]. Unique exacerbations from stressors can occur in IBD, which are psychological elements significantly contributing to symptoms worsening, illustrating potential complex gut-brain interactions [18]. This convergence shows the overlap that IBD and DGBI have, which presents a unique challenge in understanding their collective pathophysiology. 

DGBI is likely to have a multifactorial etiology, the pathophysiology of DGBI is intricate, with potential overlap between DGBI and IBD that is not fully understood. Key risk factors for symptoms related to DGBI include visceral hypersensitivity, altered gut motility, brain-gut axis dysfunction, microbiota imbalance, immune system activation from inflammation or infection, psychosocial factors, and genetic components [12,15,17]. While the exact understanding of some of these factors is incomplete, brain-gut dysfunction is well-described. Patients with DGBI often exhibit comorbid anxiety and depressive disorders, and these associations cannot be solely explained by healthcare-seeking behavior, suggesting that DGBI may be a primary manifestation of brain dysfunction [17,19-21].

The dietary habits of individuals with DGBI may contribute to the condition's pathophysiology as well. In clinical settings, many DGBI patients report associations between specific foods and their symptoms. Interestingly, these associations are not consistently reproduced in the double-blind reintroduction of suspected foods. However, dietary choices can impact the composition of the gastrointestinal microbiome, potentially influencing symptoms associated with FGID [19,20,21]. Fermentable oligo-, mono-, and disaccharides and polyols (FODMAPs) found in stone fruits, legumes, lactose-containing foods, and artificial sweeteners can exacerbate symptoms in some patients due to their fermentation and osmotic effects [22]. Another noteworthy concept is non-coeliac gluten sensitivity, where patients with DGBI experience significant symptom improvement upon gluten withdrawal despite lacking a proven diagnosis of coeliac disease [20,23].

Furthermore, a potential genetic interplay in DGBI development is intriguing, as clinical scenarios often show family clustering. Reports indicate that IBS tends to aggregate in families, with twin studies revealing higher concordance rates among monozygotic twins compared to dizygotic twins, suggesting a genetic contribution to DGBI development. Genetic components associated with immune regulation and motility cells, such as interstitial cells of Cajal, could influence gut permeability, dysmotility, and visceral hypersensitivity [1,15,20]. In addition, infection or immune regulations from infection play a crucial role in the pathophysiology of DGBI. IBS, particularly IBS-Diarrhea phenotype or other functional gastrointestinal disorders, often follows acute enteric infections. Animal studies inducing colonic inflammation through chemicals reveal a link between inflammation severity and subsequent visceral hypersensitivity, indicating a potential mechanism for symptom onset [24,25]. The gut-brain interaction is also evident, with inflammatory cytokines linked to infection or inflammation. Wouters et al. demonstrated a connection between elevated cytokine levels, anxiety scores, and DGBI symptoms, indicating that stressors can augment inflammation changes, leading to increased visceral sensitivity [20,24,26].

Overlap syndrome between IBD and DGBI and its treatment approaches and challenges

The intersection of DGBI and IBD, forming an overlap syndrome, presents a compelling and clinically significant scenario in gastroenterology. This intricate convergence challenges the conventional distinctions between organic and functional gastrointestinal disorders, creating difficulties in diagnosis and management. The shared clinical features and overlapping symptoms, such as abdominal pain, alterations in bowel habits, and irregular motility patterns, add complexity to distinguishing between DGBI and IBD [1].

Understanding the pathophysiological mechanisms of this overlap is crucial for effective patient care. The chronic inflammation characteristic of IBD may contribute to altered gut motility and visceral hypersensitivity observed in DGBI [22,26]. Conversely, the comorbid stress and anxiety associated with chronic inflammation can amplify functional symptoms, creating a cyclical relationship. This reciprocal impact underscores the need for a comprehensive approach considering both structural and functional aspects of gastrointestinal health through a holistic and multi-disciplinary strategy. In terms of diagnosis, the overlap syndrome requires a thorough evaluation, considering clinical, endoscopic, and imaging findings to achieve accuracy. This diagnostic challenge is heightened by the heterogeneity of symptoms within each condition and the potential for atypical presentations in the context of overlap.

While IBD often demands immunosuppressive and anti-inflammatory treatments to manage underlying processes, DGBI necessitates a multidimensional approach, including lifestyle modifications, dietary adjustments, and psychological interventions. The coexistence of these conditions in the overlap syndrome adds complexity, emphasizing the need for an integrated strategy addressing both inflammatory and functional aspects of gastrointestinal health. Early recognition of the overlap syndrome is crucial for improved patient outcomes and rapport and for the prevention of unnecessary investigations [27-29]. Managing the overlap syndrome requires a customized approach that addresses both the inflammatory and functional aspects of the gastrointestinal tract. Balancing pharmacological interventions for inflammation with lifestyle modifications, dietary adjustments, and psychological interventions is imperative. This multi-disciplinary approach acknowledges the intricate interplay between physical and psychological factors, aiming to optimize the quality of life for individuals grappling with the complexities of this overlap syndrome.

Addressing altered gut motility and visceral hypersensitivity often incorporates lifestyle modifications, dietary adjustments, and stress management techniques. However, the diversity of symptoms and individual responses to interventions present challenges in identifying universally effective treatments. Managing patient expectations, establishing a strong patient-clinician relationship, providing clear explanations, and connecting with the patient holistically are crucial steps that can improve acceptance and clinical outcomes [20,29].

Furthermore, when evaluating patients with persistent abdominal symptoms and potential overlap of DGBI and IBD, the initial step involves employing non-invasive markers such as C-reactive protein (CRP) and fecal calprotectin to rule out objective inflammation. It is crucial to confirm histological and endoscopic remission before diagnosing DGBI. Clinicians may inadvertently overlook DGBI, focusing solely on symptoms related to IBD, neglecting the possibility of an overlap syndrome, and potentially over-investigating [5,29].

In addition, the bidirectional influences between psychological states and gut symptoms require integrating mental health interventions and other holistic approach strategies. Examples are dietary modifications, such as the low FODMAP diet, may be helpful, and clinicians can consider fiber supplementation for diarrhea. Pharmacotherapies, including low-dose antidepressants, anti-spasmodic, and anti-motility medications, may address symptoms like abdominal pain and diarrhea [22,30,31]. Additionally, psychological therapies and physical interventions, such as cognitive-behavioral therapy (CBT), gut-directed hypnotherapy, and pelvic floor exercises, can be valuable. However, implementing these interventions may face challenges related to access and acceptance as this requires time and effect [32-39].

The coexistence of IBD and DGBI introduces complexity to treatment approaches [32,34]. Balancing the management of chronic inflammation in IBD while addressing functional symptoms requires a collaborative and multi-disciplinary effort. Clinicians must tailor interventions carefully to each patient's unique needs, considering the impact on physical and psychological well-being [5,27,34,38]. The challenge lies in optimizing symptom control without exacerbating inflammation or compromising mental health, often necessitating significant time and multiple outpatient consultations for comprehensive management of these overlapping diseases [35,40].

Emerging research directions

The dynamic landscape of IBD research is set to redefine our understanding of the disease's underlying mechanisms. Lingering questions persist regarding the specific triggers of immune dysregulation and the factors influencing the variable clinical course of the disease. Future studies may delve into unraveling the intricate genetic and environmental factors contributing to the heterogeneity of IBD manifestations. Additionally, there is a compelling need to explore the long-term impact of current therapeutic interventions and identify novel targets that could revolutionize treatment paradigms.

In the domain of DGBI, current gaps in knowledge beckon researchers to explore the nuanced relationships between psychological factors, altered gut function, and gut microbiota [34,36]. Unraveling how stress and emotional states impact gut motility and sensitivity remains a key avenue for exploration. The interaction between neurological and hormonal signals constituting the gut-brain axis provides a wealth of information on the reciprocal factors shaping symptomatology. Furthermore, emerging technologies like artificial intelligence and advanced imaging techniques could open new frontiers for studying the real-time dynamics of gut function and its modulation.

The convergence of functional gut disorders and IBD necessitates a tailored research agenda to elucidate shared pathways and distinctive features of their interplay. Exploring shared genetic markers, understanding the impact of persistent inflammation on the gut microbiome, and dissecting how psychological factors influence disease outcomes represent uncharted territories. Utilizing interdisciplinary approaches, including genomics, immunology, neurology, and psychology, will be pivotal in constructing a comprehensive understanding of the interplay between physical and psychological factors in these complex gastrointestinal conditions. Nonetheless, there is a limited evidence base for treatment options, and ongoing future research is needed to develop effective treatment paradigms [37,41].

## Conclusions

Throughout the course of this comprehensive review, we have explored the nuanced domains of chronic inflammation, altered gut motility, visceral hypersensitivity, and the bidirectional influences of psychological factors. The intersection of DGBI and IBD has surfaced as a complex realm wherein the distinctions between physical and psychological facets become blurred. The shared clinical features, overlapping symptomatology, and the intertwined impact on both disease entities highlight the intricacies inherent in their interplay. The necessity of a holistic approach becomes evident as clinicians navigate the challenges posed by chronic inflammation in IBD, the diverse spectrum of symptoms in DGBI, and the interplay between the two. Through recognizing the symbiotic relationship between the structural and functional dimensions of gastrointestinal health, clinicians, researchers, and healthcare professionals are more adept at comprehending the multifaceted nature of these conditions.

In conclusion, the call to action resonates with the need for sustained research endeavors, embracing emerging directions that pledge to reshape our comprehension of these gastrointestinal conditions. This review does not conclude here but rather transitions into a future where precision medicine, interdisciplinary collaborations, and innovative therapeutic modalities are pivotal for more targeted and effective strategies. By acknowledging the intricacies unveiled in this review, we contribute to a broader discourse that aspires to elevate the standard of care, fostering a comprehensive and compassionate approach for individuals navigating the intricate landscape of IBD and DGBI.
